# Possible contribution of endogenous carbon monoxide to the development of allergic rhinitis in guinea pigs

**DOI:** 10.1186/1476-9255-5-23

**Published:** 2008-12-05

**Authors:** Yu Shaoqing, Zhang Ruxin, Chen Yinjian, Chen Jianqiu, Zhu Chunsheng, Tang Jiangfeng, Li Genhong

**Affiliations:** 1 Department of Otolaryngology, Jinan General Hospital of PLA, Shandong, 250031, PR China; 2 Department of Otolaryngology, Huadong Hospital, Fudan University, Shanghai, 200040, PR China; 3 Department of laboratory medicine, Jinan General Hospital of PLA, Shandong, 250031, PR China

## Abstract

**Background:**

The mechanisms responsible for the development of allergic rhinitis(AR) are not fully understood. The present study was designed to explore the possible roles of carbon monoxide(CO) on the pathogenesis of AR.

**Methods:**

AR guinea pig model was established by nasal ovalbumin sensitization. Twenty-four AR guinea pigs were divided into four groups, 6 in each: Saline control group, AR sensitized group, Hemin treated group, and Zinc protoporphyrin (ZnPP) treated group. The frequency of sneezing and nose rubbing was recorded. Leukocyte infiltration in nasal lavage fluid, serum IgE level and plasma CO were measured. Expression of heme oxygenase-1 (HO-1) mRNA in nasal mucosa was determined by real time RT-PCR, and expression of HO-1 protein was detected by immunohistochemistry.

**Results:**

The frequency of sneezing and nose rubbing, leukocyte infiltration, serum IgE, plasma CO, and HO-1 mRNA levels in sensitized guinea pigs were higher than those of control (P < 0.05). Except for serum IgE level, all above parameters were even higher (P < 0.05) when treated with Hemin, a heme oxygenase-1 inducer; but significantly decreased (P < 0.05) when treated with ZnPP, a heme oxygenase inhibitor. Immunohistochemical results showed that positive staining of HO-1 was present in the lamina of mucosa of sensitized guinea pigs, and there was an increase of HO-1 immunoreactivity with Hemin administration (P < 0.05) and a decrease with ZnPP treatment.

**Conclusion:**

The endogenous CO may take part in the inflammation process of AR and is positively correlated with expression of HO-1 in nasal mucosa. Endogenous CO plays a significant role in the pathogenesis of AR.

## Background

Research on the role of the gas signal messenger such as nitric oxide (NO) and carbon monoxide (CO) in allergy medicine is a rapidly emerging field. Important roles of CO have been identified in many physiological and pathological processes relating to vasomotion, cell growth, even apoptosis [1,2]. CO is mainly produced by enzyme heme oxygenase (HO), which has been found to be expressed in almost all human tissues and organs. HO consists of three isozymes: HO-1, HO-2 and HO-3. HO-1, which is known as inducible form of heat shock protein 32 (HSP32), has been implicated in the regulation of respiratory tract inflammation [3]. HO-2 is constitutively expressed in many mammalian cells. HO-3 is also a constitutive isoform of HO. Both HO expression and CO level in the airways increase in response to hypoxic challenge and to a wide variety of inflammatory stimuli such as asthma and allergic rhinitis [4], and the expression of HO-1, but not HO-2, is upregulated in the nasal mucosa with allergic rhinitis [5].

In some airway inflammations such as asthma, induction of HO-1 may lead to highly exhaled CO concentrations, which is often observed and closely associated with chronic inflammation [6]. However, information about CO-induced effects involved in allergic rhinitis airway inflammation is rarely documented. The current study was designed to investigate the role of HO-1 in allergic rhinitis. Hemin (Ferriprotoporphyrin IX chloride), an iron containing metalloporphyrin and a substrate for HO-1, was used to increase CO level and expression of HO-1[7]. HO antagonist, zine protoporphyrin (ZnPP) was used to down-regulate HO [8]. The frequencies of sneezing and nose rubbing of guinea pigs, which represented nasal irritation, were recorded. As symbol of inflammation, levels of leukocyte infiltration in nasal lavage fluid and IgE in serum were detected. Thus, our study may facilitate a better understanding of the formation of HO-1-mediated inflammation in allergic rhinitis.

## Methods

### Materials and animal models

Twenty-four adult healthy male Hartley guinea pigs (230–280 g) were purchased from National Rodent Laboratory Animal Resources, Shanghai Branch, China. Animal models of allergic rhinitis were prepared according to the method from Al Suleimani M [9]. Guinea pigs(n = 18) were initially exposed to 1% ovalbumin (10 mg/kg, Sigma Inc.MD) in saline given as a 1% aerosol twice for 10 min each, 7 days apart. On days 14, 15 and 16, a booster of 1% ovalbumin in saline was instilled intranasally at a volume of 20 μl/nostril/day into both nostrils. On day 21 guinea pigs were challenged with 2% ovalbumin in saline instilled intranasally at a volume of 20 μl/nostril in each nostril. Eighteen guinea pigs were sensitized and divided into three groups. In the first group, animals(n = 6) were challenged intranasally with 2% ovalbumin. In the second group, named as Hemin group, animals(n = 6) were intraperitoneally administered Hemin (Sigma Inc.MD) at a dose of 10 mg/kg/day 12 h after every nose inspiration with ovalbumin and continual for two weeks. In the third group, named as ZnPP group, animals(n = 6) were intraperitoneally dministered ZnPP (Sigma Inc.MD) at a dose of 6 mg/kg/day immediately after every nose inspiration with ovalbumin and continual for two weeks too. Control animals(n = 6) were initially exposed to saline given as an aerosol twice for 10 min each at first stage, then were intranasally challenged by saline under the same conditions at a volume of 20 μl/nostril in each nostril at second stage.

### Observation of sneezing and nose rubbing and assessment of leukocyte infiltration

Frequencies of sneezing and nose rubbing were assessed as previously described by Al Suleimani M with modifications [9]. The numbers of sneezing and nose rubbing were counted for 30 min directly following nasal challenge. A sneeze was characterized by an explosive expiration just after deep inspiration. A nose rub was characterized by an external perinasal scratch with the animal's forelimbs. Nasal lavage fluid (NLF) was collected [9] at 1 h post-challenge. Nasal cavities were washed with 2 ml of pre-warmed saline infused from the tracheal side. NLF was collected from the anterior naris. Total cell count was assessed using a standard hemocytometer. Leukocytes were counted under light microscope at power 40×, using the following formula: Number of cells/ml = total number of cell counted × dilution factor × 1000/total volume counted (0.1 mm3)

### Determination of Plasma CO and serum IgE

Guinea pigs were anesthetized by intraperitoneal administration of pentobarbital (40 mg/Kg). 1 ml blood was collected from the heart through direct cardiac puncturatio, avoiding air contact. Concentration of COHb, which represented CO content in plasma, was measured using gas chromatography as described by Chalmers with spectrometer (Lambda Bio, Perkin Elmer Inc, MD)[10]. The guinea pigs were sacrificed by rapid decapitation, and blood and nasal mucosa were collected. Biopsies of nasal mucosa were taken from the inferior turbinate and put in liquid nitrogen immediately. Some of the nasal mucosa were fixed in 10% formalin for immunohistochemistry analysis. Serum IgE levels of guinea pigs were determined by ELISA methods (RB Inc.MD).

### Immunohistochemistry assay

Tissues from the specimens were fixed in 10% buffered formalin. Immunohistochemical stains were performed on formalin-fixed and paraffin-embedded 4 μm sections. The tissue sections were deparaffined, and antigen retrieval conditions included 0.1 M citrate buffer (pH 6.0) in an 800-W microwave oven for 15 minutes. The sections were incubated in 3% hydrogen peroxidase to quench endogenous tissue peroxidase for 5 minutes. The tissue sections were then incubated with a rabbit monoclonal antibody against HO-1 for 30 minutes at room temperature (1:1000 dilutions, Santa Cruz Inc.MD). The slides were stained in an automated immunostainer using a standard avidin-biotin complex staining procedure. Negative controls were performed for all cases and consisted of identically prepared slides that were treated with antibody diluents in place of primary antibody, but otherwise subjected to the same immunohistochemical staining protocol.

For quantification of HO-1 immunoreactivity, high resolution digital images were obtained from each biopsy, so that the entire mucosal area was captured. The immunoreactive score was applied for calculating the immunoreactivity of HO-1, which equaled the product of the percentage of positive cells times the average staining intensity. Percentage of positive cells was graded as follows: 0 = negative, 1 = up to 10% positive cells, 2 = 11 to 50%, 3 = 51 to 80%, 4 = >80%. Staining intensity of 0 = negative, 1 = weakly positive, 2 = moderately positive, 3 = strongly positive.

### Total RNA extraction and cDNA synthesis

Samples of nasal mucosa were shipped and stored at -80°C. They were minced with a scalpel on dry ice, transferred immediately to 2 ml polypropylene tubes, and homogenized. Total RNA was extracted using Trizol™ reagent (Invitrogen Inc, MD) following the manufacturer's instructions. The concentration and purity of RNA were determined spectrophotometrically. Then the synthesis of cDNA was performed according to a cDNA synthesis kit (PrimeScript RTase, TaKaRa Inc, Japan).

### Real time Reverse Transcriptase-Polymerase Chain Reaction (RT-PCR) for HO-1 mRNA Expression

**Figure 1:**
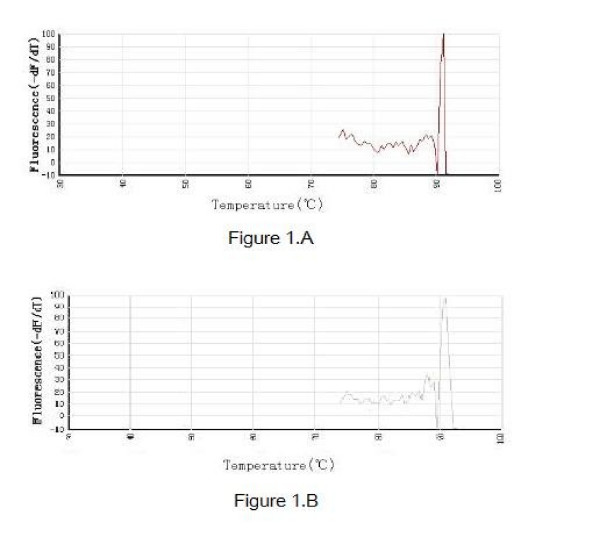
**The typical amplification and melting curves of real-time RT-PCR for HO-1 (A) and β-actin(B)**. The figure shows a sharp peak with a melting temperature of HO-1 of 92°C and β-actin of 90°C.

**Figure 2:**
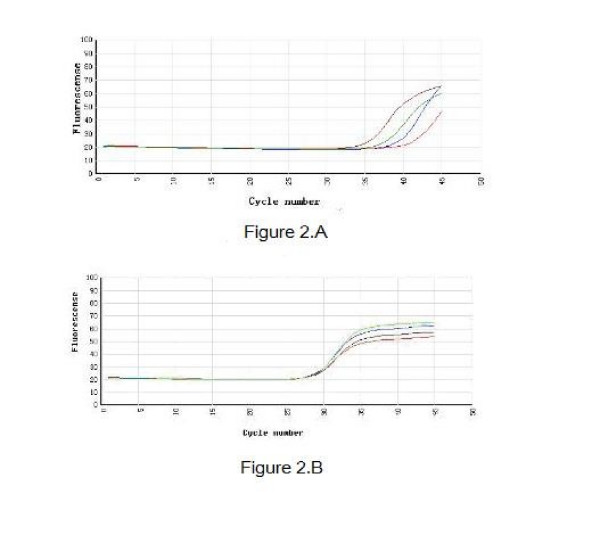
**Cycles of HO-1 (A) and β-actin (B)**. The vertical axis represents the degree of amplification by SYBR-Green fluorescence and the horizontal axis represents the number of amplification cycles. Fig 2A:With 45 cycles, the groups have different amplification of HO-1. For the four curves from left to right: Hemin group, AR group, ZnPP group, control group. It indicate the amounts of PCR pruducts were Hemin group > AR group > ZnPP group > control group. Fig 2B:With the same cycle number, the groups have similar amplification of β-actin.

To determine the expression of the HO-1 gene in nasal mucosa, fluorescent quantitative real time RT-PCR assay was performed. The sequences of the primers (TaKaRa Inc, Japan) specific for HO-1 were performed with sense (GAAGGAGGCCACCAAGGAGG) and antisense (AGGTCACCCAGGTAGCG GGA) primers, with an expected size of the amplified sequence of 370 bp. β-actin was used as control (sense: ACCCTTAAGGCCAACCGTGAAAAG, antisense: TCATGAGGTAG TCTGTCAGGT, 240 bp). Then the incubation of cDNA and primer was performed at 95°C for 5 min, and the PCR reaction proceeded for 45 cycles: 95°C for 20 s, 57°C for 20 s, and 72°C for 20 s, and a final incubation at 72°C for 7 min in a programmable thermal cycler (Line-Gene real-time PCR detection system, bioer Inc, China) using a thermostable Taq DNA polymerase (SYBR PrimeScript Ex Taq, TaKaRa Inc, Japan). Fluorescent product was measured by a single acquisition at 86°C after each cycle. After the completion of PCR amplification, a melting curve analysis was performed. Fig. 1 shows a sharp peak with a melting temperature (Tm) of HO-1 of 92°C and β-actin of 90°C. For each sample, the amounts of the target and control (β-actin, a housekeeping gene) were determined. The typical amplification curves of real-time RT-PCR for HO-1 and β-actin mRNA are shown in Fig. 2. The amount of the target was then divided by the amount of the endogenous reference, to obtain a normalized target value. The PCR products were visualized and photographed under ultraviolet light by staining with ethidium bromide on 1.5% agarose gels.

### Statistics

All data were expressed as mean ± S.D. Statistical analyses of data were performed using ANOVA for multiple comparison and LSD for comparison among groups, and Pearson Correlation for the two-variable correlation analysis. P < 0.05 was considered to be statistically significant.

## Results

### Concentration of COHb in plasma

**Figure 3:**
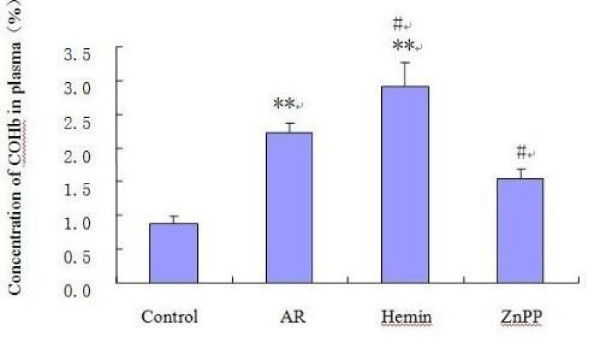
**The blood COHb level of groups**. Each column and vertical bar represents the mean ± S.D. *,**: Significantly different from the control group (p < 0.05 and p < 0.01, respectively). #,## Significantly different from the AR group (p < 0.05 and p < 0.01, respectively).

The plasma COHb level of AR group was higher than that of non-sensitized group (p < 0.01), which confirmed the results of previous research. COHb level increased significantly after being treated with Hemin, and decreased after ZnPP administration as compared with AR group (p < 0.05) (Fig. 3).

### Sneezing, nose rubbing and leukocyte infiltration

**Figure 4:**
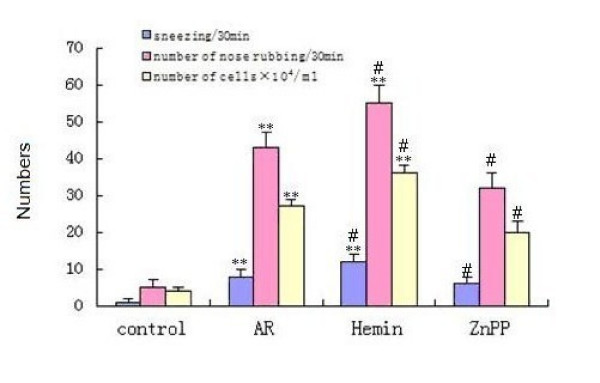
**The Sneezing, nose rubbing and leukocyte infiltration of guinea pigs**. Each column and vertical bar represents the mean ± S.D. *,**: Significantly different from the control group (p < 0.05 and p < 0.01, respectively). #,## Significantly different from the AR group (p < 0.05 and p < 0.01, respectively).

Sneezing frequency and number of nose rubbing in sensitized AR group were significantly increased (p < 0.01) as compared with those in non-sensitized group, and increased further in Hemin treated group as compared with those in AR group(p < 0.05), but significantly decreased in ZnPP treated group(p < 0.05) (Fig. 4). In AR group, there was a significant increase of total cell count in NLF (p < 0.01), especially eosinophil and neutrophil as compared with those in non-sensitized groups. Total cell count significantly increased after Hemin treatment and decreased after ZnPP treatment (p < 0.05) as compared with AR group.

### Change of IgE in serum

**Figure 5:**
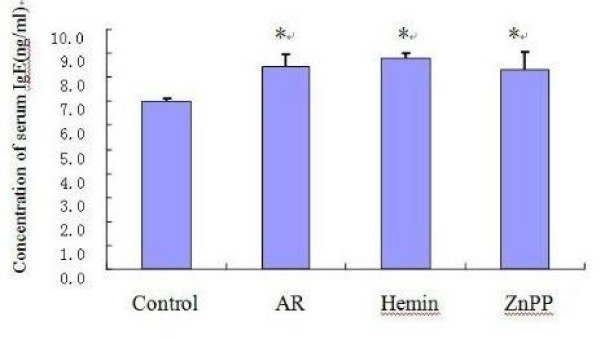
**The serum IgE level**. Each column and vertical bar represents the mean ± S.D. *,**: Significantly different from the control group (p < 0.05 and p < 0.01, respectively). #,## Significantly different from the AR group (p < 0.05 and p < 0.01, respectively).

Serum IgE levels in sensitized AR groups were significantly increased (p < 0.05) as compared with those in non-sensitized group. but no significant differences of IgE were observed among groups of AR, Hemin and ZnPP(p > 0.05) (Fig. 5).

### Immunohistochemical staining for HO-1

**Figure 6:**
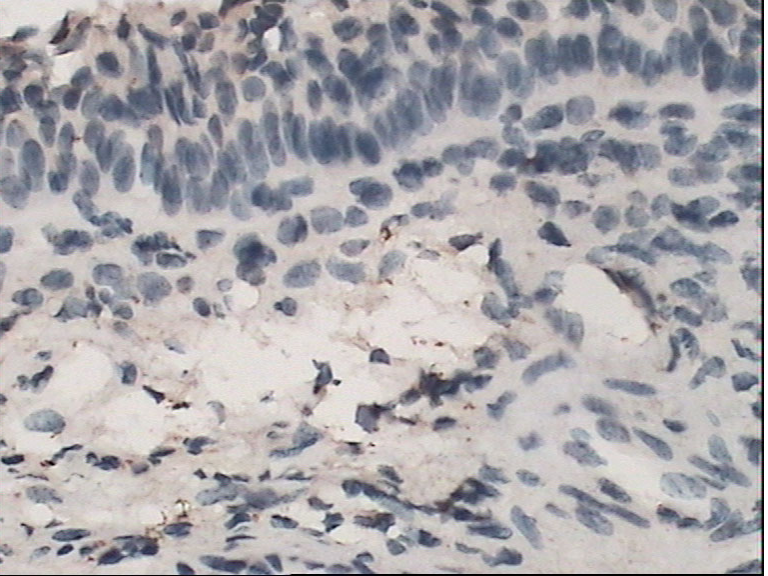
**Immunoreactivity of HO-1 in nasal mucosa**. Immunoreactivity of HO-1 in normal nasal mucosa. No positive staining was seen for HO-1. (original magnification × 40).

**Figure 7:**
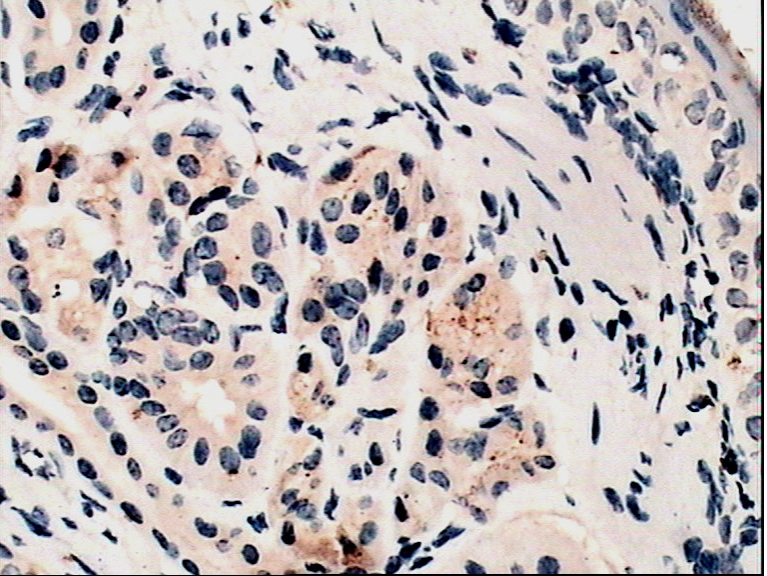
**Immunoreactivity of HO-1 in nasal mucosa**. Immunoreactivity of HO-1 in allergic nasal mucosa: Cytoplasmic staining (brown) is seen in seromucous glands, mesenchymal cells and inflammatory cells of the lamina propria. (original magnification × 40).

**Figure 8:**
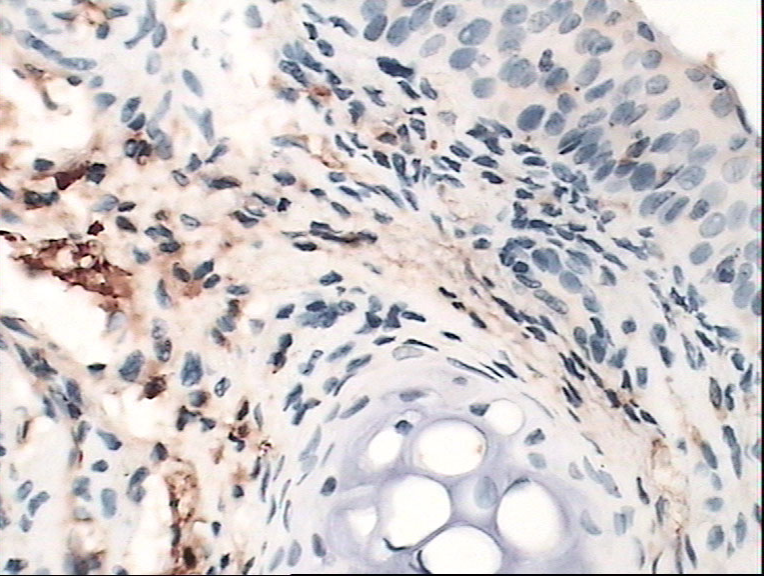
**Immunoreactivity of HO-1 in nasal mucosa**. Positive staining also can be seen in cytoplasm of vascular endothelial cells, but no HO-1 expression was found in respiratory epithelium and cartilage. (original magnification × 40).

**Figure 9:**
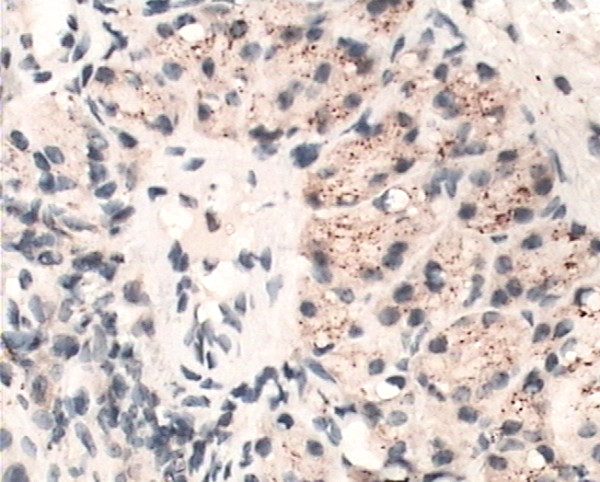
**Immunoreactivity of HO-1 in nasal mucosa**. Immunoreactivity of HO-1 in Hemin group nasal mucosa: A particularly intense staining was present in epithelium cells of seromucous glands. (original magnification × 40).

**Figure 10:**
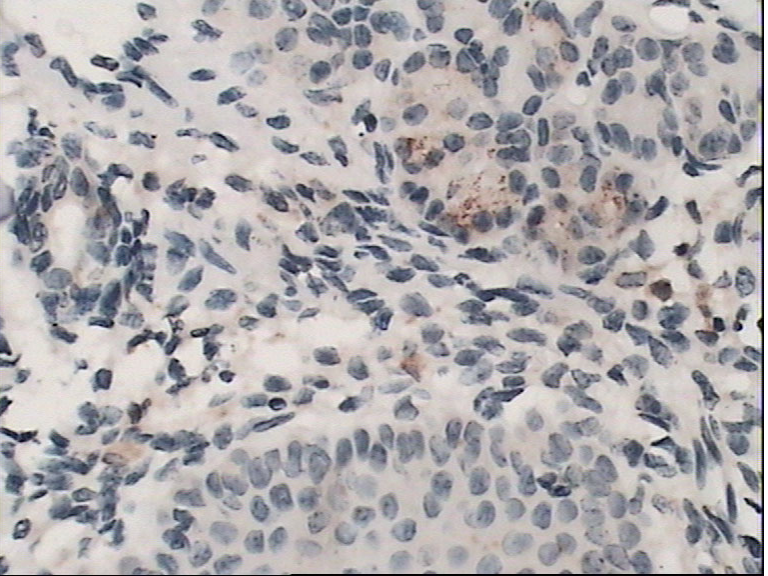
**Immunoreactivity of HO-1 in nasal mucosa**. Immunoreactivity of HO-1 in ZnPP group nasal mucosa: Weak expression of HO-1 was observed in lamina of mucosa cells. (original magnification × 40).

The yellow-brown cytoplasm represented positive signals of HO-1 expression. Positive granules were not observed in the control group(Fig. 6) but found expressed in allergic nasal mucosa, and those were distributed mainly in the cytoplasm of seromucous glands, mesenchymal cells and inflammatory cells in lamina (Fig. 7), which. Cytoplasmic staining was also seen in endothelial cells lining dilated vessels. No HO-1 expression was found in respiratory epithelium and cartilage in either group (Fig. 8). Negative control sections gave no detectable staining. Strongly positive staining was present in nasal mucosa of Hemin group and weak expression of HO-1 was observed in lamina of mucosa of ZnPP group (Fig. 9 and 10).

The total immunoreactivity for HO-1, in relation to the area of mucosal tissue, was 0.50 ± 0.54 in controls (n = 6), 3.33 ± 0.81 in guinea pigs after allergen challenge (n = 6), 5.00 ± 2.19 in sensitized guinea pigs after Hemin administration (n = 6), and 1.83 ± 0.75 in ZnPP treated group (n = 6). There was an increase in HO-1 immunoreactivity after allergen challenge (p = 0.01), and an increase further in HO-1 immunoreactivity after Hemin administration (p < 0.05). However, there was a decrease in HO-1 immunoreactivity after ZnPP treatment (p < 0.05).

### Expression of HO-1 by real-time RT-PCR

**Figure 11:**
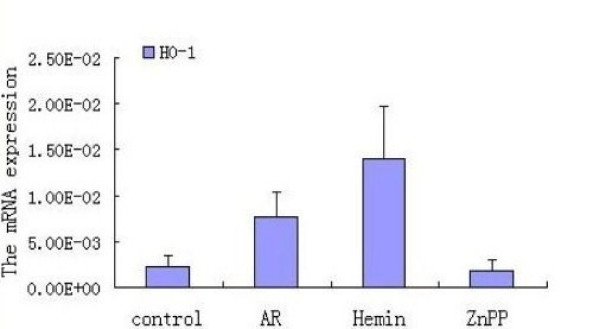
**Expression of HO-1 mRNA**. Each column and vertical bar represents the mean ± S.D. *,**: Significantly different from the control group (p < 0.05 and p < 0.01, respectively). #,## Significantly different from the AR group (p < 0.05 and p < 0.01, respectively).

**Figure 12:**
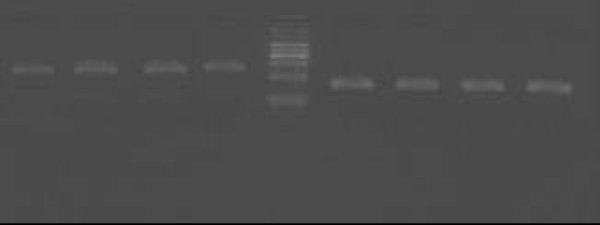
**Image of gel of RT-PCR for HO-1 (*left*) and β-actin (*right*) mRNA from nasal mucosa of guinea pigs**. Sizes of PCR products are 370 bp(HO-1) and 240 bp(β-actin). Lanes 1–4 were products of HO-1 of normal, AR, Hemin, ZnPP groups, and lanes 6–9 were products of β-actin (housekeeping gene), and lane 5 was a DNA marker to mark the size of the PCR product(100 600 bp). There was an increase in HO-1 mRNA in AR group compared with control, and HO-1 mRNA increased after Hemin treated and decreased after ZnPP treated, whereas there was no change in β-actin mRNA.

**Figure 13:**
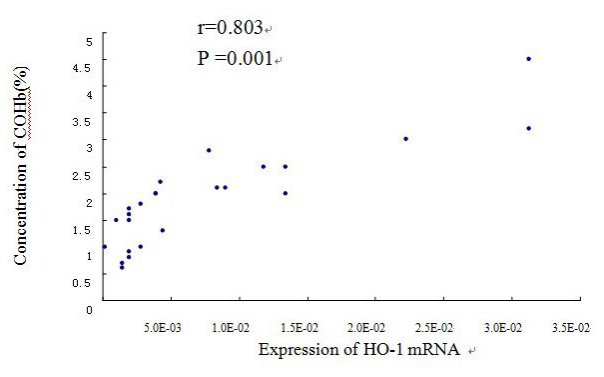
**Correlation between concentration of COHb and HO-1 mRNA expression level**. Pearson Correlation was used to analyze the relationship between the level of HO-1 mRNA and concentration of COHb. There was a high significant direct relationship between them (r = 0.803, P = 0.001).

The cumulative data for mRNA expression of HO-1 is presented in Fig. 11. HO-1 mRNA expression was upregulated in AR group as compared with control (p < 0.05), and the expression was further increased after being stimulated with Hemin (p < 0.05), whereas it was inhibited by ZnPP (p < 0.05). To verify the melting curve results, representative samples of the PCR products were run on 1.5% agarose gels. Electrophoresis result showed that the order of HO-1 mRNA expression levels from high to low was Hemin treated group, AR sensitized group, control group and ZnPP treated group in turn (Fig. 12). Moreover, a significant correlation between HO-1 mRNA expression and the concentration of COHb was found (r = 0.803, P = 0.001) (Fig. 13). It suggested that level of CO was regulated by HO-1 in a concentration-dependent manner, and Hemin and ZnPP might affect the level of CO by regulating the expression of HO-1 in AR.

## Discussion

This study shows that expression of HO-1 and concentration of COHb increased in sensitized guinea pigs, which suggested that raised levels of CO in serum of AR animals were associated with increased expression of HO-1 in nasal mucosa. Therefore, the endogenous CO may take part in the pathogenic process of AR and is positively correlated with expression of HO-1 in nasal mucosa.

Positive HO-1 expression can be seen in the cytoplasm of seromucous glands, mesenchymal cells and inflammatory cells in lamina, but not in the nasal respiratory epithelium of sensitized guinea pigs, which confirms early observation of Elhini A [5]. HO-1 up-regulation in seromucous glands within the submucosal layer suggests that HO-1 may play a role locally at submucosal level rather than epithelial level. Positive expression also can be seen in cytoplasm of vascular endothelial cells. In the research of Pitkin L [11], immunohistochemistry for HO-1 had revealed distinctly positive staining in vascular endothelial cells and erythrocytes in nasal mucosa of allergic rhinitis patient. Up-regulation of local HO-1 production in allergic nasal submucosa indicates that CO may be involved in the inflammatory process of allergic rhinitis at the submucosal level, and perhaps contribute to the increase of nasal irrigation. Increased expression of HO-1 in submucosal layer of nasal mucosa of Hemin treated group is likely to be related to the oversupply of substrate. HO-1 antagonist ZnPP attenuated the expression of HO-1 in nasal mucosa. These results indicated that Hemin and ZnPP successfully influenced immunoreactivity of HO-1 in nasal mucosa.

This study is the first to show that HO-1 expression in nasal mucosa was up-regulated by Hemin in vivo in allergic rhinitis, accompanied by increased sneezing frequency and number of nose rubbing. There was a marked increase in total cell count (p < 0.01) in lavage fluid especially eosinophil and neutrophil. Hemin increases level of plasma CO and number of infiltrating cells via HO-1 induction [12,13]. Cellular levels of free heme are approximately 100 nM or less. Higher amounts can be injurious, and kidney and other organs can be injured by increased amounts of heme from heme proteins[14], so low concentration of Hemin was chosen and only continual used for two weeks. Research of Andersson JA had shown that low concentrations of hemin (10^-11 ^m to 10^-9 ^m) enhanced the migratory response of neutrophils, whereas higher concentrations (10^-7 ^m to 10^-5 ^m) inhibited migration. In this study, both plasma CO level and HO-1 expression of guinea pigs were significantly increased after Hemin treated, even more eosinophil and neutrophil were induced accompanied by aggravation of symptoms of AR [15]. ZnPP as an analog of heme can play a prominent role in the catabolism of heme and cause decreased HO activity and CO formation. Administration of ZnPP to rats leads to its deposition in a variety of organs (plasma, liver, spleen, kidney, lung, and brain), causing decreased HO activity and CO formation[16]. Our findings also showed that HO-1 inhibitor ZnPP suppressed the expression of HO-1 of nasal mocusa and level of CO in vivo. In our study, ZnPP attenuated airway hyperreactivity including decrease of sneezing frequency, nose rubbing, and inhibition of inflammatory cell infiltration.

CO can serve as intracellular and intercellular signaling molecules similar to NO. It is able to bind to iron atom of the heme moiety associated with soluble guanylate cyclase (sGC), thereby increasing intracellular cGMP product. This result leads to protein phosphorylation and ion channels regulation, and finally to cell relaxation accompanied by the outflow of K^+ ^increase and the influx of Ca^2+ ^reduce[17,18]. It also can activate the protein kinase G (PKG) and affect the protein kinase A (PKA) activity and KCa channel, resulting in the inhibition of Ca^2+ ^influx of vascular smooth muscle cells to reduce the intracellular Ca^2+ ^concentration, and further leads to the vasodilatation[19].

CO may have pro-inflammatory effects since it is also a potent vasodilator and may increase plasma exudation from airway vessels [20]. CO can elicit important physiological responses like bronchial relaxation and vasodilation in asthma [21]. However, the anti-inflammatory effect of endogenous CO is closely related to the expression of HO-1, and expression of HO-1 has been described as a marker of response to oxidative stress. The oxidant stress associated with HO-1 upregulation in the ovalbumin-challenged guinea pigs could contribute to an induction in the number of neutrophils, eosinophils, and lymphocytes in the nasal cavity. HO-1 is induced in various cells inferior to subepithelial region such as vascular endothelial cells, gland cells, infiltrating inflammatory cells in the nasal mucosa. The induction of HO-1 has been implicated as an antioxidant defense mechanism and has been associated with inflammation of airway disease include adult respiratory distress syndrome, oxidant-induced lung injury, and chronic inflammatory disorders such as chronic obstructive pulmonary disease and asthma [22-24].

The inducible HO-1 catalyzes the rate-limiting step of heme oxidation to biliverdin, carbon monoxide (CO), and iron. Apart from the physiological role of CO, other metabolites of HO, such as bilirubin, acts as an antioxidant directly by its peroxyl radical-scavenging properties as well as by decreasing inflammatory cell recruitment during airway inflammation[25]; and the other metabolic product, such as ferrous ion, also acts as an potent antioxidant for cell protection [26].

There was a significant increase of serum IgE level in AR group as compared with control. The increase of IgE level during allergic reactions has long been recognized as an important step in immediate hypersensitivity reactions to antigen. High serum IgE levels also can be seen in hemin and ZnPP treated group, but without significant changes as compared with ovalbumin-sensitized guinea pigs (p > 0.05). The results indicate that endogenous CO takes part in the pathogenesis of AR mainly through regulating cGMP production, affecting vascular dilation, glandular secretion, and cytokines or chemokines migration, rather than influencing the IgE-mediated immune response process. However, such a hypothesis has not yet been elucidated and further research is needed.

## Conclusion

Findings of this study implicate a direct involvement of CO in the inflammatory process of allergic rhinitis and the effect of CO may attribute to the action of HO-1. HO-1 acts as an important modulator of the inflammatory response in upper airway in allergic rhinitis. Understanding of these mechanisms is essential for future therapeutic strategies and the successful treatment of the allergic rhinitis.

## Competing interests

The authors declare that they have no competing interests.

## Authors' contributions

YSQ and ZRX designed and performed the study. Culturing and stimulation of animals as well as evaluating experimental data was done by LGH and TJF. CJQ and ZCS quantified plasma CO and IgE levels. Immunohistochemical staining was done by YSQ. CYJ designed and performed the study of RT-PCR and was involved with interpretation of results. All authors read and approved the final manuscript.
